# Grand Rapids Veterinary College

**Published:** 1900-05

**Authors:** 


### COMMENCEMENT EXERCISES.

### GRAND RAPIDS VETERINARY COLLEGE.

This College held its annual commencement exercises in the College Auditorium, Thursday, March 29. They were attended by a large number of the leading veterinarians of the State, among whom were George Hare, President, and W. W. Thorburn, Secretary of the Michigan State Veterinary Board of Examiners. The graduating class consisted of the following: J. B. Weir, Smicksburg, Pa.; T. P. Pomeroy, Freeport, Mich.; A. E. McCall, Memphis, Mich.; F. O. N. Hovey, Coopersville, Mich., and H. L. Hickox, city. Coleman Nockolds, V.S., M.D., having completed a post-graduate course, was granted the degree of D.V.S. Weir and Pomeroy received honorable reward for high attainment in their college work. Hovey and McCall received honorable mention for second and third places. In the junior class C. E. Dornheim, Providence, R. I., took first; A. B. Warrener, Portsmouth, Ohio, second, and G. S. Throp, third prize in anatomy. In medicine, B. F. Baldwin, first; C. E. Dornheim, second, and A. B. Warrener, third.

One of the most pleasant features of the exercises was the conferring of the honorary degree, doctor of veterinary science, upon W. W. Thorburn, Secretary of the Michigan State Veterinary Board of Examiners. This was done by the President of the Board of Trustees, Professor J. A. McPherson, in a few well-chosen remarks, followed by the college dean, who at some length went over the work performed by Dr. Thorburn and the legislative fight that has been waged for years in this State between the American and foreign element. He showed from statistics that Dr. Thorburn had done more work and devoted a great deal more time and attention to this work than any other veterinarian in the State, and did it without price. He said that Governor Pingree should be applauded by every American citizen who has the welfare of the veterinary profession at heart for his recognition of the doctor, by appointing him to a place on the State Veterinary Board; and that the trustees and faculty of the college have but fittingly, through this act, expressed their gratitude to the Governor for this appointment. Dr. Thorburn said that the conferring of the degree was entirely unexpected, and feelingly acknowledged the honor paid him by the trustees of the college, and wishing the college many years of prosperity.

---

